# A New Species of *Ascodipteron* (Diptera: Hippoboscidae) from China Based on Morphology and DNA Barcodes †

**DOI:** 10.3390/insects13121148

**Published:** 2022-12-13

**Authors:** Haoran Sun, Liang Ding, Thomas Pape, Dong Zhang

**Affiliations:** 1 School of Ecology and Nature Conservation, Beijing Forestry University, Qinghua East Road 35, Beijing 100083, China; 2 Go with KIDS Natural History Workshop, Beijing 100032, China; 3 Natural History Museum of Denmark, Science Faculty, University of Copenhagen, 2100 Copenhagen, Denmark

**Keywords:** description, *Ascodipteron*, bat fly, endoparasite

## Abstract

**Simple Summary:**

*Ascodipteron* Adensamer, 1896 is a small genus of bat flies distributed in tropical and subtropical areas of the Eastern Hemisphere, with 16 valid species currently known. *Ascodipteron* has not been taxonomically well-studied in China, where only four species have been reported. Here, a new species, *Ascodipteron guoliangi* sp. nov. from Fujian Province, is described, based on dealate neosomic females and supported by molecular data from the cytochrome B (*Cytb*) and cytochrome oxidase subunit I (*COI*) genes.

**Abstract:**

A new species of the genus *Ascodipteron* Adensamer, 1896 (Diptera: Hippoboscidae) is described from Fujian, namely *A*. *guoliangi* sp. nov. Habitus and diagnostic details, as well as the attachment sites on the host, are documented with photographs. A detailed comparison of the new species with related species is provided and the new species is accommodated in the most recent key to the world species of *Ascodipteron*.

## 1. Introduction

*Ascodipteron* Adensamer, 1896 is a small genus belonging to the strebline grade of nycteribiine Hippoboscidae [1,2] and currently comprising 16 described species from tropical and subtropical areas of the Eastern Hemisphere [3,4]. Ascodipterine bat flies are highly specialized subcutaneous endoparasites, easily distinguishable from other streblines by their unusual mode of parasitic life and strong polymorphism in the adult stage, where females lose halteres, wings, and legs at the trochanter immediately after attachment to a host bat and transform into neosomes [3,4,5]. The neosome produces a considerable swelling in the bat’s skin, but the only part visible externally is the posterior, knob-like tip of the abdomen [6,7]. Thus, these bat parasites have received attention from but a few entomologists.

South and Southeast China belong to the Oriental region and is an area known for its high biodiversity [8], but very few studies have been undertaken on the dipterous parasites of bats. According to present knowledge, only four species of *Ascodipteron* have been recorded in China [3,4], and in this study we describe a new species of *Ascodipteron* from Fujian, *Ascodipteron guoliangi* sp. nov., with extensive documentation of its dealate neosomic females, and it is incorporated in a key to the known species of the genus.

## 2. Materials and Methods

### 2.1. Specimen Collection and Preparation

Nine bat flies were collected from a colony of the East Asian tailless leaf-nosed bat, *Coelops frithii* Blyth, 1848 in May 2021, roosting in a small, abandoned bomb shelter at 011 Township Road, Chenda Town, Sanyuan District, Sanming, Fujian, China (26°23′38.28″ N, 117°34′16.00″ E; 303 m above sea level). An additional three ascodipterine specimens collected between May 2020 and January 2022 were used for DNA sequence analysis. Details of all specimens are provided in Table 1.

Entire female ascodipterine bat flies (neosomes) were removed with forceps without hurting the host. All specimens were preserved in 95% ethanol and deposited at the Museum of Beijing Forestry University, Beijing, China (MBFU).

### 2.2. Specimen Imaging, Measurements, and Terminology

Z-stack photographs were acquired with a Zeiss Axio Zoom.V16 microscope (Carl Zeiss AG, Oberkochen, Germany) equipped with a PlanApo Z 1.0×/0.25 FWD 60 objective and an AxioCam 503 color camera. Images were processed with the software Zen 2 (Carl Zeiss AG, Oberkochen, Germany) and Adobe Photoshop 2021 (Adobe Systems Incorporated, San Jose, USA) by cropping, contrast enhancement, and removal of the background.

Photographs were taken with EF 100 mm f/2.8L IS USM and MP-E 65 mm f/2.8 1–5X lenses attached to a Canon 5D Mark IV SLR camera. Images and plates were processed on a standard Windows 10 platform using Adobe Photoshop 2021 (Adobe Systems, Inc., San Jose, CA, USA).

**Figure 1:**
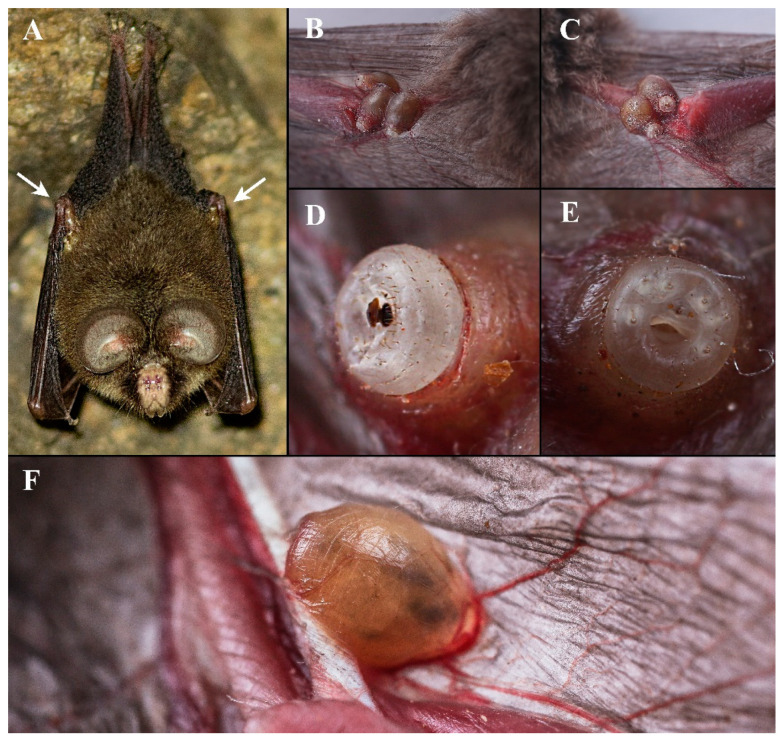
*Ascodipteron guoliangi* sp. nov. and its host *Coelops frithii*. (**A**). Roosting host with neosomes in each elbow pit (arrows). (**B**,**C**). Neosomes embedded in host tissue. (**D**,**E**). Neosome terminalia protruding from host tissue. (**F**). Blood vessels passing through the neosome. (1A: courtesy Mr. Huang Yue (Nanjing, China).)

**Figure 2:**
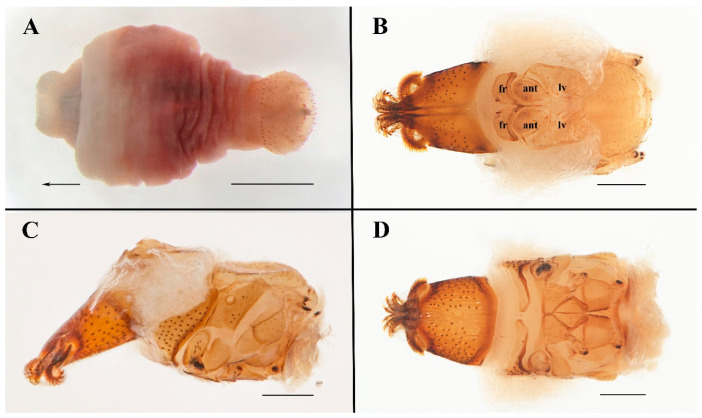
*Ascodipteron guoliangi* sp. nov., ex. *Coelops frithii*, China. (**A**). Whole neosome (head and thorax fully withdrawn, arrow indicates direction of the head) (BFU-2434, neosome holotype). (**B**–**D**). Head and thorax; dorsal view (BFU–2437, neosome paratype) (**B**), lateral view (**C**), and ventral view (**D**). Abbreviations: ant—antenna; fr—frons; and lv—lateral vertex. Scale bars: **A** = 500 μm; **B**,**C** = 200 μm.

Measurements and terminology follow Hastriter and Bush (2006) [5], except that the term “genital aperture” has been replaced with “terminalia”. This term refers to the knob-like tip of the neosome abdomen that protrudes from the pouch or warble formed by the host (Figure 1D,E), and which is morphologically distinct from the remaining part of the abdomen (Figure 2A), and as such it conforms to contemporary terminology [9]. The terminalia are comprised of segments V–VII with the often diagnostic circular arrangements of setae plus the cerci, anus, and vaginal orifice.

### 2.3. DNA Extraction, Amplification, Sequencing and Sequence Editing

Two specimens (BFU-2435, BFU-2436) of *Ascodipteron guoliangi* sp. nov., four specimens of *A*. sp2, six of *A*. *sanmingense* Sun et al., and two of *A*. *speiserianum* Muir were dissected to extract total genomic DNA using the TIANamp Genomic DNA Kit (Tiangen, Beijing, China). Head and thorax were used for all specimens, except for two of *A*. *sanmingense*, which hatched from a puparium, and for which the whole body was used. After extraction, the head and thorax or whole body were cleaned with demineralized water and retained with the remaining body parts as vouchers, deposited in the Museum of Beijing Forestry University, Beijing, China (MBFU). The mitochondrial cytochrome B (*Cytb*) gene was amplified using the primer pair A5 (forward: 5′-AGG RCA AAT ATC ATT TTG AG-3′) and B1.1 (reverse: 5′-AAA TAT CAT TCT GGT TGA ATA TG-3′) [10]. The mitochondrial cytochrome oxidase subunit I (*COI*) gene was amplified and sequenced using primers LCO1490: GGT CAA CAA ATC ATA AAG ATA TTG G and HCO2198: TAA ACT TCA GGG TGA CCA AAA AAT CA [11]. PCR reactions were conducted as described in Zhang et al. (2016) [12] and Yan et al. (2019) [13], and amplification conditions were as described by Dittmar and Whiting (2003) [10] and Zhang et al. (2013) [14]. The PCR products were purified and sequenced bidirectionally by BGI Inc., Beijing, China.

SeqMan Pro v. 7.1.0 (DNASTAR Inc., Maddison, WI, USA) was used to edit and assemble the forward and reverse sequences.

### 2.4. DNA Sequence Analysis

The only three cytochrome b gene (*Cytb*) sequences of the genus *Ascodipteron* were downloaded from GenBank. The *COI* and *Cytb* sequences in this study, together with the *Cytb* sequences from GenBank, were aligned using Muscle as implemented in Mega X [15,16]. Subsequently, nucleotide sequence divergences were calculated, using the Kimura 2-parameter (K2P) model in Mega X.

## 3. Results

### 3.1. Taxonomy

*Ascodipteron guoliangi* sp. nov.

LSID. urn:lsid:zoobank.org:act: E5946FCF-F557-47A6-81AC-EBB577BB48F1

**Material examined.** Holotype ♀ (dealate), China: Fujian, Sanming, Sanyuan District, Chenda Town, Road no. 011, ex. *Coelops frithii*, in the elbow pit where the upper arm meets the forearm, 12.V.2021, H.R. Sun and L. Ding, complete whole neosome (BFU-2434) (MBFU). 

Paratypes 5 ♀ (dealate), same data as holotype (BFU-2522–2526) (MBFU); 2 ♀ (dealate), same data as holotype but dissected and sequenced, with head, thorax, terminalia and gut preserved (BFU-2435–2436) (MBFU); 1 ♀ (dealate), same data as holotype but dissected, all parts were preserved (BFU-2522–2526) (MBFU).

**Diagnosis.***Ascodipteron guoliangi* sp. nov. is most similar to *A. phyllorhinae* Adensamer and *A. sanmingense*. It can be separated from the former by the setae on the labial theca: ca. 50+, peg-like, spiniform setae dorsally and 100+ uniform setae ventrally in *A. guoliangi* sp. nov., versus 18–20 peg-like, spiniform setae dorsally and 46–50 uniform setae ventrally in *A. phyllorhinae*, and from both by R4 absent (present in *A. phyllorhinae* and *A. sanmingense*).

**Figure 3:**
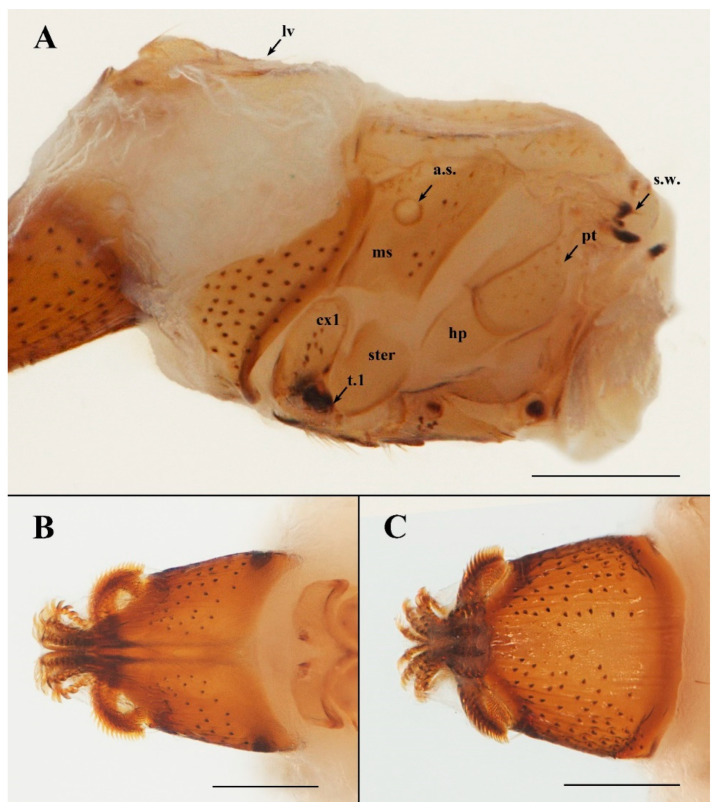
*Ascodipteron guoliangi* sp. nov., ex. *Coelops. frithii*, China (BFU-2437, neosome paratype). (**A**). Thorax, lateral view. (**B**). Labial theca, dorsal view. (**C**). Labial theca, ventral view. Abbreviations: a.s.—anterior thoracic spiracle; cx1—coxa 1; g—gena; hp—hypopleuron; lv—lateral vertex; ms—mesopleuron; pt—pteropleuron; s.w.—stump of wing; ster—sternopleuron; t.1—trochanter 1. Scale bars: **A**–**C** = 200 μm.

**Description. Female.** Anterior part of abdomen pyriform, the posterior part mushroom-shaped. **Head**. Length and width of labial theca nearly equal (Figure 2B,D and Figure 3B,C); posterior margin concave dorsally, convex ventrally. Labial theca dorsally with ca. 50+ peg-like, spiniform setae and ventrally with ca. 100+ similar setae. Peg-like setae identical to those on gena. Gena with ca. 40 irregularly scattered peg-like setae on dorsal half, anterior margin convex and posterior margin concave, approach triangular (Figure 3A). 

**Figure 4:**
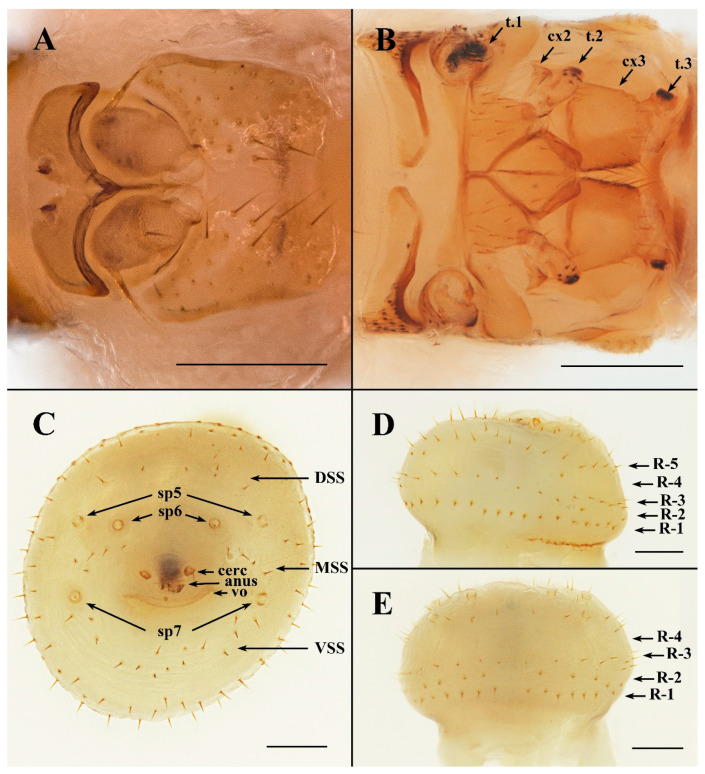
*Ascodipteron guoliangi* sp. nov., ex. *Coelops. frithii*, China (BFU-2437, neosome paratype). (**A**). Frons and lateral vertex. (**B**). Thorax, ventral view. (**C**). Terminalia, posterior view. (**D**,**E**). Terminalia, ventral view (**D**) and dorsal view (**E**), indicating five terminal annular rows of setae (R4 absent, R5 only in ventral view). Abbreviations: cerc—cercus; cx1–3—coxa 1–3; DSS—dorsal spiracular setae; MSS—medial spiracular setae; R1–5—abdominal setae arranged roughly into annular rows comprised of variable types of setae, R1 the proximal and R5 the distal row; sp5–sp7—spiracles 5–7; t.1–3—trochanter 1–3; vo—vaginal orifice; VSS—ventral spiracular setae. Scale bars: **A**,**B** = 100 μm; **C**,**D** = 200 μm.

Frons excised and bifurcated at middle of anterior margin, thick and blunt at lateral margins. Arista with multiple fine branches, basal antennal segment with single long seta. Lateral vertex with concave anteromedial margin; adorned with 22–30 long thin setae on each side; less than twice as long as wide, with slight longitudinal fold in lateral portion of sclerite outer margin (Figure 4A). Two anterior lobes of occipital sclerite rounded, central anterior margin broadly concave. **Thorax** (Figure 3A). Scutum with numerous long setae, devoid of setae along mid-line. Mesopleuron with 4–8 sharp, nonpigmented papilla-like setae anterior to large round spiracle; setae posterior to spiracle of three varieties: 7–10 short, peg-like, spiniform setae; 1–4 long, slender setae (medial); 2–6 longer, sharp, spiniform setae (along posterior margin and dorsal). Pteropleuron with 15–17 nonpigmented papilla-like setae in dorsal half of sclerite; ventral half devoid of setae. Hypopleuron and sternopleuron without setae. Coxa 1 with 8–11 pigmented, peg-like, spiniform setae and 2–5 long, slender setae. Coxa 2 with 2–4 long, slender setae. Coxa 3 with cluster of 15–19 long, slender setae. Trochanters 1 and 2 each with 4–6 min, spiniform setae on anterior apex; 1–2 slender setae on posteroapical margin. Trochanter 3 with 5–8 slender setae. Prosternum devoid of setae, mesosternum with 20–24 slender setae, metasternum with 8–12 slender setae. **Terminalia** (Figure 4C–E). R1 and R2 with short, thick setae; R3 and R5 with thin, longer setae, R4 absent and R5 only found ventrally. VSS (ventral spiracular setae situated in ventral, arching row or grouping between spiracles 7; Figure 4C) with 12–15 long setae. MSS (medial spiracular setae with paired symmetrical groups situated between spiracles 6 and 7; Figure 4C) with 3–5 seta. DSS (dorsal spiracular setae situated in single dorsal arching row between spiracles 5; Figure 4C) with dorsally arched row of six long setae. Each cercus very small, flat, with two long and a few minute setae. Diameter of cercus ca. 36 μm (n = 4, range: 33–38 μm). **Dimensions.** Head and thorax: 1251 μm (n = 3, range: 1198–1278 μm); labial theca, length: 527 μm (n = 3, range: 514–535 μm), width: 376 μm (n = 3, range: 368–388 μm); terminalia, diameter: 983 μm (n = 3, range: 975–993 μm); neosome, length: 3417 μm (n = 2, range: 3281–3552 μm).

**Male.**

Unknown.

**Etymology.**

The new species is named in honor of Mr. Guo Liang (Fuzhou, China), an enthusiastic amateur entomologist who discovered the cave and thereby was instrumental to affording the first author the possibility to collect valuable specimens. The eponym is also a tribute to Mr. Guo Liang’s decades of dedication to collecting and investigating the insects of Fujian Province.

**Distribution.**

Oriental—China (Fujian).

**Remarks.***Ascodipteron guoliangi* sp. nov. is only known from China (Fujian). Its known host, *C. frithiii*, is considered to be relatively widespread in South Asia, but it is listed as ‘near threatened’ in the IUCN Red List [17].

*Ascodipteron guoliangi* sp. nov. will run to couplet 14 in the identification key to dealate ascodipterine females proposed by Hastriter (2007) [3], and it can be incorporated in the key together with the recently described *A*. *sanmingense* as follows, with host data given in square brackets:

[Couplets 1–12 as in Hastriter (2007) [3]]
13 Spiniform setae on labial theca large and pigmented. MSS comprised two or more setae. R1 may or may not be present ...........................................................................................14
– Spiniform setae smaller and not pigmented; MSS comprised one seta, or none .........17
14 R1–R5 present and complete .................................................................................................15
– R1–R5 absent or incomplete .................................................................................................16
15 Labial theca dorsally with 25–30 lightly pigmented peg-like spiniform setae. Lateral vertex without fold or reinforcement in lateral portion of sclerite. [*Hipposideros* spp., usually on wing, SE China to Solomon Islands.].......*A. phyllorhinae* Adensamer, 1896
– Labial theca dorsally with ca. 50+ normally pigmented peg-like spiniform setae. Lateral vertex with longitudinal fold or reinforcement in the lateral portion of the sclerite. [*Hipposideros armiger*, at base of ear or on lower jaw area, SE China.]....................................................................................*A. sanmingense* Sun, Ding, Yan, Pape & Zhang, 2021
16 First abdominal annular row (R1) present; fourth abdominal annular row (R4) absent. [*Coelops frithii*, in elbow pit where upper arm meets forearm, SE China.]..................................................................................................................*Ascodipteron guoliangi* sp. nov.
– First abdominal annular row (R1) absent; fourth abdominal annular row (R4) present. [*Rhinolophus* spp., Africa.]....................................................................*A. brevior* Maa, 1965
17 MSS comprised of one seta. R1 and R2 absent (*M. schreibersi*, Africa).............................................................................................................................................*A. theodori* Maa, 1965
– MSS lacking setae. R1–R3 present, with short, triangular spines (*M. schreibersi*, Africa)..........................................................................................................................................*A. minor* Theodor, 1973

### 3.2. Biology

Muir (1912) [6], Jobling (1939) [7], Dick and Patterson (2006) [1], and Hastriter (2007) [3] provided extensive biological information for ascodipterines, with differences between species mainly including host and attachment sites. *Ascodipteron guoliangi* sp. nov. has so far only been recovered embedded in the elbow pit where the upper arm meets the forearm of *Coelops frithii* (Figure 1A–C). A position in the elbow pit may reduce the risk of host grooming and facilitate access to blood vessels suitable for feeding (Figure 1F), while allowing unimpeded breathing when the bat rests in the roost (Figure 1A). Each neosome supports a thin layer of host skin, forming a bulge of the same shape as the neosome. Its flask-shaped body is smooth, with a very distinct constriction between the terminalia and the major, pyriform part of the abdomen, which lies inside the skin of the bat (Figure 1D,E). *Ascodipteron guoliangi* sp. nov. is the only *Ascodipteron* known to parasitize *Coelops frithii*. Five neosomes on a single host is the most we have ever seen, three on one forearm and two on the other, packed close together (Figure 1A–C).

**Figure 5:**
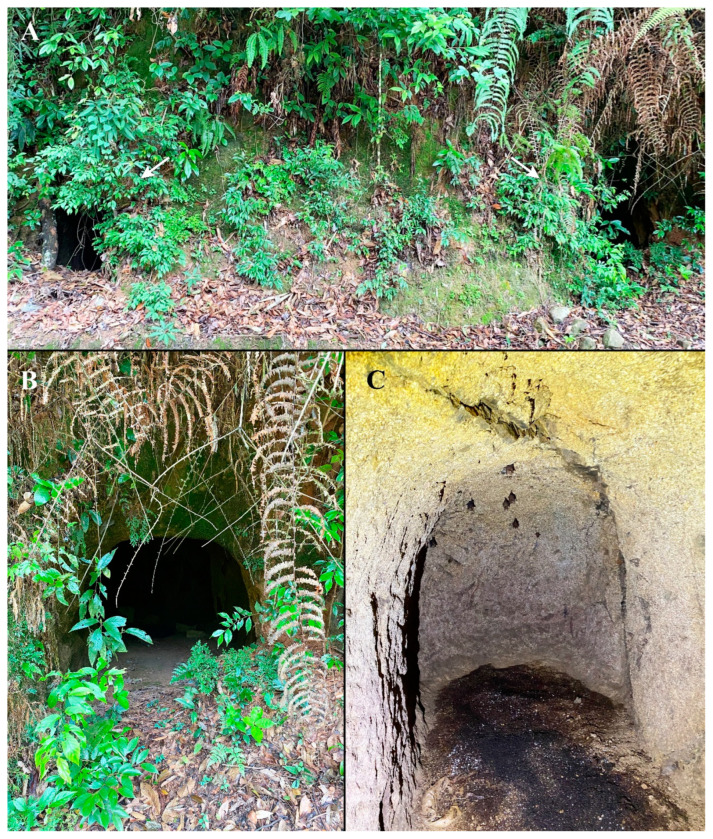
Habitat of *Coelops frithii*. (**A**). The roadside slope with the two openings (white arrows) to the cave (a former bomb shelter). (**B**). The opening to the right branch of the cave. (**C**). The deepest part of the right cave.

The cave where *C. frithii* was found is an abandoned bomb shelter located in a hilly area at the outskirts of Sanming, Fujian Province, China. The cave was dug into the slope along a gravel road, and it has two entrances to two short tunnels, which meet and then branch out again, the cave thereby taking the shape of an X. Each branch is about 1.3 m high and 1 m wide and with a depth of 3–4 m (Figure 5A,B).

The cave is slightly damp, but there is no water seepage. The soil is a mixture of quartzite fragments and sandstone, and the cave floor is covered with accumulated bat droppings. During the present study, the cave was a habitat for 4–5 species of bats with about fifty individuals, of which only five or six were *C. frithii*, roosting together on the roof of the deepest parts of the cave (Figure 5C).

### 3.3. Molecular Results

A 384 bp fragment of *Cytb* was sequenced from 11 *Ascodipteron* specimens containing five species. Pairwise comparison of the fragments gave an average genetic divergence of 4.90% between *A. guoliangi* sp. nov. and A. sp2, 6.31% between *A. guoliangi* sp. nov. and *A. phyllorhinae*, 9.77% between *A. guoliangi* sp. nov. and *A. sanmingense*, 13.20% between *A. guoliangi* sp. nov. and *A. speiserianum*, and 12.29% between *A. guoliangi* sp. nov. and *A.* sp2 (Table 2).

A 672 bp fragment of COI was sequenced from 13 *Ascodipteron* specimens containing four species. Pairwise comparison of the fragments gave an average genetic divergence of 5.00% between *A. guoliangi* sp. nov. and *A.* sp2, 6.61% between *A. guoliangi* sp. nov. and *A. sanmingense*, and 8.42% between *A. guoliangi* sp. nov. and *A. speiserianum* (Table 3).

The intraspecific variation of all known ascodipterine *Cytb* and *COI* sequences are 0.00–0.79% (Table 2) and 0.00–1.45% (Table 3), respectively. Nucleotide sequence data were deposited in the GenBank database under the accession numbers indicated in Table 1.

## 4. Discussion

The present record of *A. guoliangi* sp. nov. is the first documentation of a bat fly from the Southeast Asian genus *Coelops* Blyth, 1848. *Coelops* belongs to the bat family Hipposideridae and contains two species, the widely distributed *C. frithii* (East Asian tailless leaf-nosed bat) and the more narrowly distributed *C. robinsoni* (Bonhote, 1908) (Malayan tailless leaf-nosed bat), from which no bat flies have been recorded. Of the six genera included in the family Hipposideridae, species of *Ascodipteron* have previously been recorded exclusively from *Hipposideros* Gray, 1831, although at least the genus *Aselliscus* Tate, 1941 has been examined for ectoparasites [5]. Current evidence indicates that bat flies are highly host specific [18], and species of *Ascodipteron* appear to be rather specific in the selection of attachment site [3,18,19]. The specimens of *A. guoliangi* sp. nov. obtained in the present study were all embedded in the elbow pit, and although several species of *Ascodipteron* are recorded for an attachment site on the wing, there are no records specifically from the elbow [3].

## Figures and Tables

**Table 1:** Specimens with GenBank accession numbers and host and locality. Superscript numbers behind binomial names indicate different specimens of the same species, from different hosts and/or habitats. Sequences downloaded from GenBank marked by asterisks. ”-” indicates no data.

	Species	GenBank Accession Numbers		Host	Locality
		*Cytb*	*COI*		
1	*Ascodipteron guoliangi* sp. nov. ^1^	-	OP900074	*Coelops frithii*	Chenda Town, Sanyuan District, Sanming, Fujian, China (in abandoned bomb shelter)
2	*Ascodipteron guoliangi* sp. nov. ^2^	OP903228	OP900075	*Coelops frithii*	Chenda Town, Sanyuan District, Sanming, Fujian, China (in abandoned bomb shelter)
3	*Ascodipteron* sp2 ^1^	-	OP900076	*Hipposideros pratti*	Jingzhou, Huaihua, Hunan, China (in cave)
4	*Ascodipteron* sp2 ^2^	-	OP900077	*Hipposideros pratti*	Jingzhou, Huaihua, Hunan, China (in cave)
5	*Ascodipteron* sp2 ^3^	OP903229	OP900078	*Hipposideros pratti*	Jingzhou, Huaihua, Hunan, China (in cave)
6	*Ascodipteron* sp2 ^4^	OP903230	OP900079	*Hipposideros pratti*	Jingzhou, Huaihua, Hunan, China (in cave)
7	*Ascodipteron sanmingense*^1^ Sun, Ding, Yan, Pape and Zhang, 2021	OP903231	OP900080	*Hipposideros armiger*	Sanming, Fujian, China (in abandoned bomb shelter)
8	*Ascodipteron sanmingense* ^2^	-	OP900081	*Hipposideros armiger*	Tianmushan, Hangzhou, Zhejiang, China (in cave)
9	*Ascodipteron sanmingense* ^3^	OP903232	OP900082	hatched from a puparium	Chenda Town, Sanyuan District, Sanming, Fujian, China (in abandoned bomb shelter)
10	*Ascodipteron sanmingense* ^4^	OP903233	-	hatched from a puparium	Chenda Town, Sanyuan District, Sanming, Fujian, China (in abandoned bomb shelter)
11	*Ascodipteron sanmingense* ^5^	-	OP900083	*Hipposideros armiger*	Qunying Second Village, Meilie District, Sanming, Fujian, China (in abandoned bomb shelter)
12	*Ascodipteron sanmingense* ^6^	-	OP900084	*Hipposideros armiger*	Qunying Second Village, Meilie District, Sanming, Fujian, China (in abandoned bomb shelter)
13	*Ascodipteron sanmingense* *	MW822598.1	-	*Hipposideros armiger*	Qunying Second Village, Meilie District, Sanming, Fujian, China (in abandoned bomb shelter)
14	*Ascodipteron speiserianum*^1^ Muir, 1912	OP903234	OP900085	*Miniopterus* sp.	Xianren cave, Haikou, Hainan, China
15	*Ascodipteron speiserianum* ^2^	OP903235	OP900086	*Miniopterus* sp.	Xianren cave, Haikou, Hainan, China
16	*Ascodipteron phyllorhinae* * Adensamer, 1896	DQ133149.1	-	*Hipposiderus bicolor*	Tiger Cave, Penang, Malaysia
17	*Ascodipteron* n. sp. *	DQ133154.1	-	*Hipposiderus bicolor*	Tiger Cave, Penang, Malaysia

**Table 2:** Pairwise differences of mitochondrial cytochrome b gene (*Cytb*) sequences between species, based on Kimura 2-parameter. Numbers in column headers refer to species and populations listed in their respective rows. Superscript numbers behind binomial names indicate different specimens of the same species, from different hosts and/or habitats. Sequences downloaded from GenBank marked by asterisks. All specimens are neosomes obtained from a host except number 7, which is a male that emerged from puparium.

		1	2	3	4	5	6	7	8	9	10
1	*Ascodipteron guoliangi* sp. nov. ^2^										
2	*Ascodipteron* sp2 ^3^	0.0490									
3	*Ascodipteron* sp2 ^4^	0.0490	0.0000								
4	*Ascodipteron phyllorhinae* *	0.0631	0.0279	0.0279							
5	*Ascodipteron sanmingense* ^1^	0.1011	0.0831	0.0831	0.0811						
6	*Ascodipteron sanmingense* ^3^	0.0980	0.0801	0.0801	0.0811	0.0079					
7	*Ascodipteron sanmingense* ^4^	0.0980	0.0801	0.0801	0.0780	0.0026	0.0052				
8	*Ascodipteron sanmingense* *	0.0937	0.0751	0.0751	0.0780	0.0027	0.0027	0.0000			
9	*Ascodipteron speiserianum* ^1^	0.1320	0.1164	0.1164	0.1093	0.1192	0.1224	0.1224	0.1223		
10	*Ascodipteron speiserianum* ^2^	0.1320	0.1164	0.1164	0.1093	0.1192	0.1224	0.1224	0.1223	0.0000	
11	*Ascodipteron* sp. *	0.1229	0.1096	0.1096	0.1063	0.1227	0.1160	0.1193	0.1193	0.0914	0.0914

**Table 3:** Pairwise differences of cytochrome oxidase subunit I gene (*COI*) sequences between species, based on Kimura 2-parameter. Numbers in column headers refer to species and populations listed in their respective rows. Superscript numbers behind binomial names indicate different specimens of the same species, from different hosts and/or habitats. All specimens are neosomes obtained from host except number 9, which emerged from puparium.

		1	2	3	4	5	6	7	8	9	10	11	12
1	*Ascodipteron guoliangi* sp. nov. ^1^												
2	*Ascodipteron guoliangi* sp. nov. ^2^	0.0063											
3	*Ascodipteron* sp2 ^1^	0.0504	0.0523										
4	*Ascodipteron* sp2 ^2^	0.0494	0.0521	0.0113									
5	*Ascodipteron* sp2 ^3^	0.0510	0.0477	0.0084	0.0137								
6	*Ascodipteron* sp2 ^4^	0.0510	0.0461	0.0084	0.0120	0.0000							
7	*Ascodipteron sanmingense* ^1^	0.0636	0.0655	0.0614	0.0655	0.0584	0.0584						
8	*Ascodipteron sanmingense* ^2^	0.0732	0.0750	0.0629	0.0706	0.0628	0.0628	0.0145					
9	*Ascodipteron sanmingense* ^3^	0.0611	0.0611	0.0590	0.0565	0.0495	0.0495	0.0036	0.0107				
10	*Ascodipteron sanmingense* ^5^	0.0626	0.0645	0.0533	0.0593	0.0533	0.0533	0.0034	0.0120	0.0000			
11	*Ascodipteron sanmingense* ^6^	0.0664	0.0683	0.0570	0.0635	0.0570	0.0570	0.0000	0.0120	0.0036	0.0034		
12	*Ascodipteron speiserianum* ^1^	0.0849	0.0802	0.0721	0.0887	0.0778	0.0761	0.0876	0.0925	0.0726	0.0765	0.0808	
13	*Ascodipteron speiserianum* ^2^	0.0872	0.0845	0.0833	0.0851	0.0826	0.0826	0.0847	0.0905	0.0724	0.0754	0.0793	0.0034

## Data Availability

The data generated in this study are provided here, and they are also available upon request from the corresponding author.

## Abbreviations

ant	antenna
a.s.	anterior thoracic spiracle
cerc	cercus
cx1–3	coxa 1–3
fr	frons
g	gena
hp	hypopleuron
lv	lateral vertex
ms	mesopleuron
pt	pteropleuron
s.w.	stump of wing
ster	sternopleuron
sp5–7	spiracles 5–7
t.1–3	trochanter 1–3
vo	vaginal orifice
DSS	dorsal spiracular setae
MSS	medial spiracular setae
VSS	ventral spiracular setae
R1–5	abdominal setae arranged roughly into annular rows, R1 the proximal and R5 the distal row
